# Evaluation of Psoas Muscle Atrophy and the Degree of Fat Infiltration After Unilateral Hip Arthroplasty

**DOI:** 10.7759/cureus.41506

**Published:** 2023-07-07

**Authors:** Ahmet Serhat Genc, Anil Agar, Nizamettin Güzel

**Affiliations:** 1 Orthopedics and Traumatology Department, Samsun Training and Research Hospital, Samsun, TUR; 2 Orthopedics and Traumatology Department, Saglik Bilimleri University, Kanuni Sultan Suleyman Training and Research Hospital, Istanbul, TUR

**Keywords:** hip joint, fat intensity, psoas muscle atrophy, hips, arthroplasty

## Abstract

Objective: Atrophy of the muscles around the hip and thigh has been reported in patients with hip osteoarthritis (OA). Total hip arthroplasty (THA) reduces pain and improves quality of life and activity levels. Muscle strength of the hip and thigh also improves after THA. This study aimed to determine whether there is significant psoas muscle atrophy and to evaluate the degree of fat infiltration after unilateral hip arthroplasty.

Subject and methods: Patients who underwent unilateral total hip arthroplasty for primary hip osteoarthritis and who had lumbar vertebra magnetic resonance imaging (MRI) for any reason in the one-year preoperative and postoperative period were evaluated retrospectively. The degree of fat infiltration was also graded visually based on a modified Goutallier rating system.

Results: The study was conducted with a total of 58 patients aged between 38 and 75, including 15 males and 43 females. Compared to the preoperative psoas muscle area values on the operated sides of the patients participating in the study, the decrease in the postoperative psoas muscle area was found to be statistically significant (p:0.000; p<0.05). Furthermore, the decrease in psoas muscle area on the non-operated side of the patients was also statistically significant (p:0.000; p<0.05). There was also a positive correlation between preoperative and postoperative psoas muscle areas (p:0.000; p<0.05).

Conclusion: Early identification of psoas muscle mass reduction may allow for a more proactive psoas strength improvement program to improve post-operative function and mobility.

## Introduction

One of the most effective and common orthopedic operations is total hip arthroplasty (THA), which is recognized as the standard treatment for severe hip joint deterioration. Patients with hip osteoarthritis (OA) have been noted to have atrophy of the muscles surrounding the hip and thigh [1-3]. Total hip arthroplasty (THA) lowers pain while enhancing activity levels and quality of life. Following THA, the muscle strength in the hip and thigh also increases. Few papers evaluate changes in hip and thigh muscle volumes after THA objectively [1,4].

It is well established that cross-sectional area mostly determines muscular strength [5]. Since it is believed that muscle cross-sectional area reflects muscle volume, it may serve as a potential biomarker of muscular atrophy. Additionally, a measure of muscle quality was determined by the amount of fat infiltration in the muscle. Goutallier has previously shown that the clinical result of rotator cuff restoration surgery is negatively correlated with infraspinatus muscle fat infiltration [6]. Therefore, it is thought that factors influencing muscle strength include the cross-sectional size of the muscle and the level of fat infiltration in the muscle, both of which have been demonstrated to rise with aging [7]. Hip osteoarthritis has been associated with a decrease in the cross-sectional area of hip muscles and an increase in fat infiltration [1,4,8]. According to what was discovered [4], these changes lasted for two years following a total hip replacement. Other research, however, found that the gluteal and thigh muscles had increased muscle volume for over two years after total hip replacement (THR) in a larger patient cohort [9]. Despite this research, little is known about the ways in which the psoas muscle alters following hip replacement surgery. The psoas muscle, which is the main hip flexor, is essential for actions like walking, ascending stairs, descending stairs, and standing up from a chair.

Our hypothesis in this study is that there is an increase in psoas muscle volume due to increased mobility after total hip replacement in patients with coxarthrosis who already have atrophy of the psoas muscle. The aim of this study was to evaluate the degree of fat infiltration and determine whether there is significant psoas muscle atrophy following unilateral hip arthroplasty.

## Materials and methods

Approval for this retrospective study was granted by the Institutional Ethics Committee (SUKAEK-2023, 8/27). The current study was conducted in line with the 2008 amendment to the 1975 Declaration of Helsinki and the institutional and national responsible committees for human experimentation's ethical requirements. Because it was retrospective, informed consent was not required.

A retrospective analysis of patients who underwent total hip arthroplasty between January 2018 and January 2020 in hospital was performed. First of all, patients who underwent unilateral THA were scanned from the hospital archive system. No age limit was applied to the screening. Patients who underwent unilateral THA for primary hip osteoarthritis and who had lumbar vertebra magnetic resonance imaging (MRI) for any reason in the one-year preoperative and one-year postoperative periods were included in the study. The following patients were not included in the study: those with systemic diseases of the nervous system and muscles, congenital or childhood hip disease, any history of hip trauma, surgery, inflammatory joint disease, tumors, or low back injury following a lower extremity or total hip arthroplasty operation.

Patients who applied for surface replacement arthroplasty and disclosed a significant lifetime history of low back pain that necessitated inactivity, inquiry, or therapy were also disqualified. Patients who utilized walking aids and engaged in unilateral sports were also disqualified.

**Figure 1:**
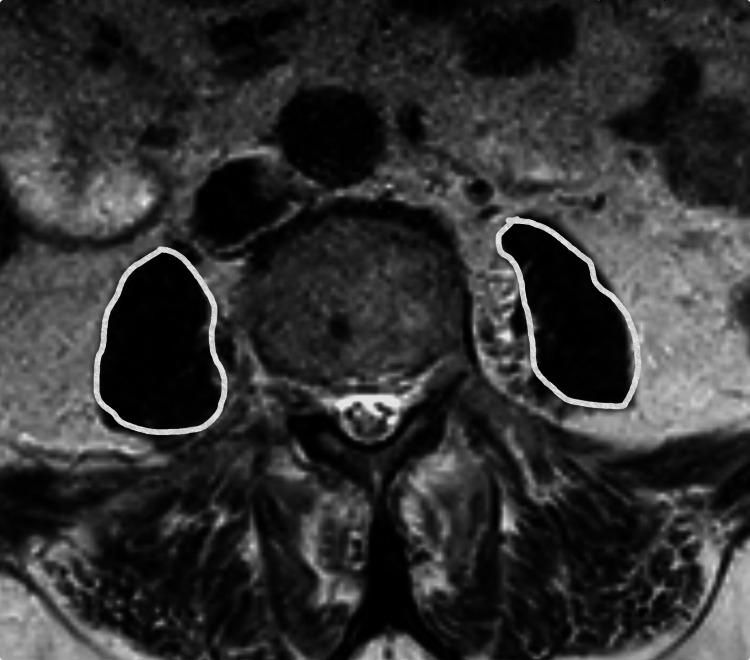
MR image used in measurements. The area marked in white shows the psoas muscle.

All patients were operated on with a posterior approach, and all patients received the same hip implant. All surgical procedures were performed by the senior author. Only initial scans of patients with multiple post-operative MRI lumbar spine scans (Figure 1) were included in the main results.

A radiologist with three years of experience completed the patients' MRI interpretations. For the evaluation, axial T2-weighted pictures at the level of the L3-4 intervertebral disc were used.

By drawing a line along the border of the muscles, the cross-sectional area of the psoas muscles on either side was computed. A modified Goutallier rating system was used to visually rate the level of fat infiltration. This categorization approach is based on preliminary information from a CT scan of the rotator cuff, even though it has been applied to evaluate other muscle groups on MRI [6,10]. The bilateral psoas muscle area and fat infiltration at the level of the L3/4 intervertebral disc were evaluated using preoperative and postoperative lumbar MRI imaging.

Statistics

The IBM Corp. Released 2013. IBM SPSS Statistics for Windows, Version 22.0. Armonk, NY: IBM Corp. application was utilized for statistical analysis when analyzing the study's findings. The Shapiro-Wilks test was used to assess the study data's compliance with the parameters' normal distribution. The Wilcoxon signed rank test was used to compare quantitative data within groups, non-normally distributed variables, and descriptive statistical techniques (mean, standard deviation, and frequency). The associations between parameters that did not follow the normal distribution were investigated using Spearman's rho correlation analysis. The level of significance used for evaluation was 0.05.

## Results

After screening the patients, it was seen that there were a total of 92 patients. After applying the exclusion criteria, the study was conducted with a total of 58 patients aged between 38 and 75, including 15 males and 43 females. Twenty-eight (48.3%) of the cases were operated on the right side, and 30 (51.7%) were operated on the left side (Table 1).

**Table 1:** Measurement of preoperative and postoperative Goutallier classification *p<0.05; Min: minimum; Max: maximum; SS: standard deviation

		Operated Side		Total
		Right	Left	
		(Min-Max)-(Mean±SS (median))	(Min-Max)-(Mean±SS (median))	(Min-Max)-(Mean±SS (median))
Grade-right	Preoperative	(0-1)-(0.32±0.48(0))	(0-0)-(0±0(0))	(0-1)-(0.16±0.37(0))
	Postoperative	(0-2)-(0.86±0.65(1))	(0-0)-(0±0(0))	(0-2)-(0.41±0.62(0))
	P	0.002*	1.000	
Grade-left	Preoperative	(0-1)-(0.04±0.19(0))	(0-1)-(0.17±0.38(0))	(0-1)-(0.1±0.31(0))
	Postoperative	(0-0)-(0±0(0))	(0-2)-(1.03±0.67(1))	(0-2)-(0.53±0.71(0))
	p	0.317	0.000*	

Compared to the preoperative psoas muscle area values on the operated sides of the patients participating in the study, the decrease in the postoperative psoas muscle area was found to be statistically significant (p:0.000; p<0.05). Furthermore, the decrease in psoas muscle area on the non-operated side of the patients was also statistically significant (p:0.000; p<0.05) (Table 1).

When the patients were evaluated according to the Goutallier classification, which evaluates the fatty infiltration of the psoas muscle, a statistically significant increase was found in the postoperative grade compared to the preoperative grade (p:0.000; p<0.05); however, no statistically significant change was observed in the fatty infiltration grade on the non-operated side (p>0.05) (Table 2).

**Table 2:** Measurement of preoperative and postoperative psoas muscle areas *p<0.05; Min: minimum; Max: maximum; SS: standard deviation

		Operated Side		Total
		Right	Left	
		(Min-Max)-(Mean±SS (median))	(Min-Max)-(Mean±SS (median))	(Min-Max)-(Mean±SS (median))
Psoas muscle area-right	Preoperative	(3626-10951)-(6360.14±1876.04(5921.5))	(3043-12014)-(5626.5±2240.71(5715.5))	(3043-12014)-(5980.67±2087.66(5815.5))
	Postoperative	(3028-12774)-(5311.18±2382.94(4444))	(4063-14654)-(6642.63±2618.2(6613.5))	(3028-14654)-(5999.86±2574.45(5082))
	p	0.001*	0.000*	
Psoas muscle area-left	Preoperative	(4014-12974)-(6018.25±2433.58(5036.5))	(3268-10525)-(5535.93±1701.04(5604))	(3268-12974)-(5768.78±2082.44(5466.5))
	Postoperative	(4214-14600)-(7046.86±2525.38(6399.5))	(3010-11074)-(4747.27±1988.26(4311))	(3010-14600)-(5857.41±2525.04(5032))
	P	0.000*	0.000*	

**Figure 2:**
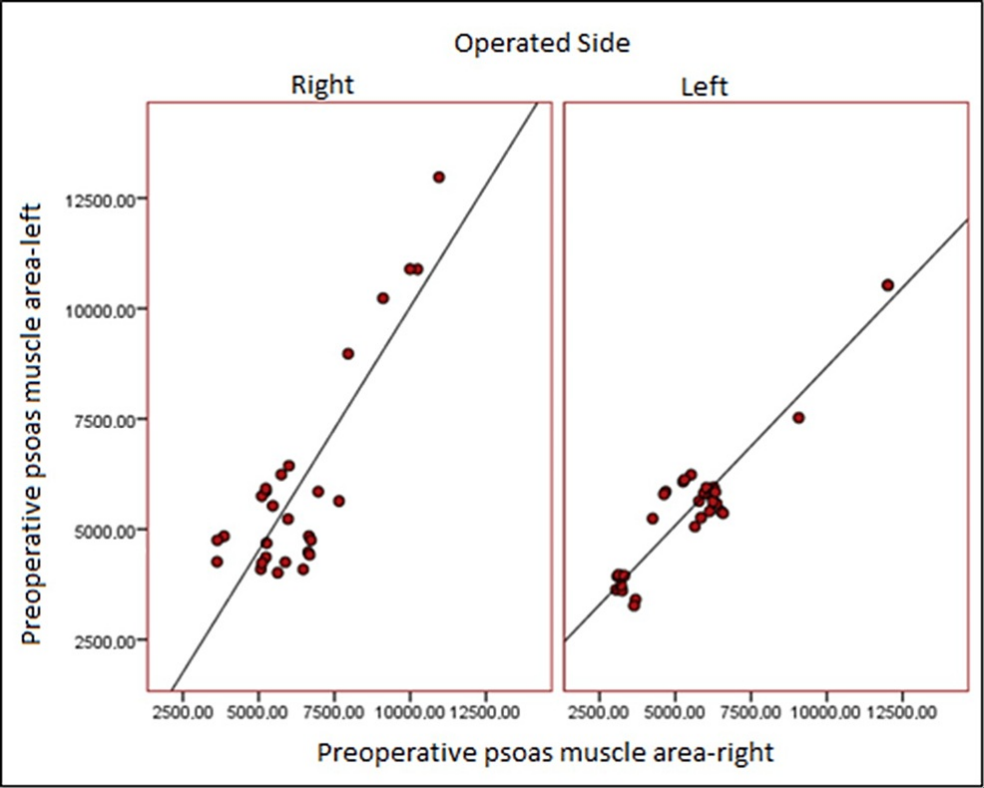
Distribution of preoperative psoas muscle area

**Figure 3:**
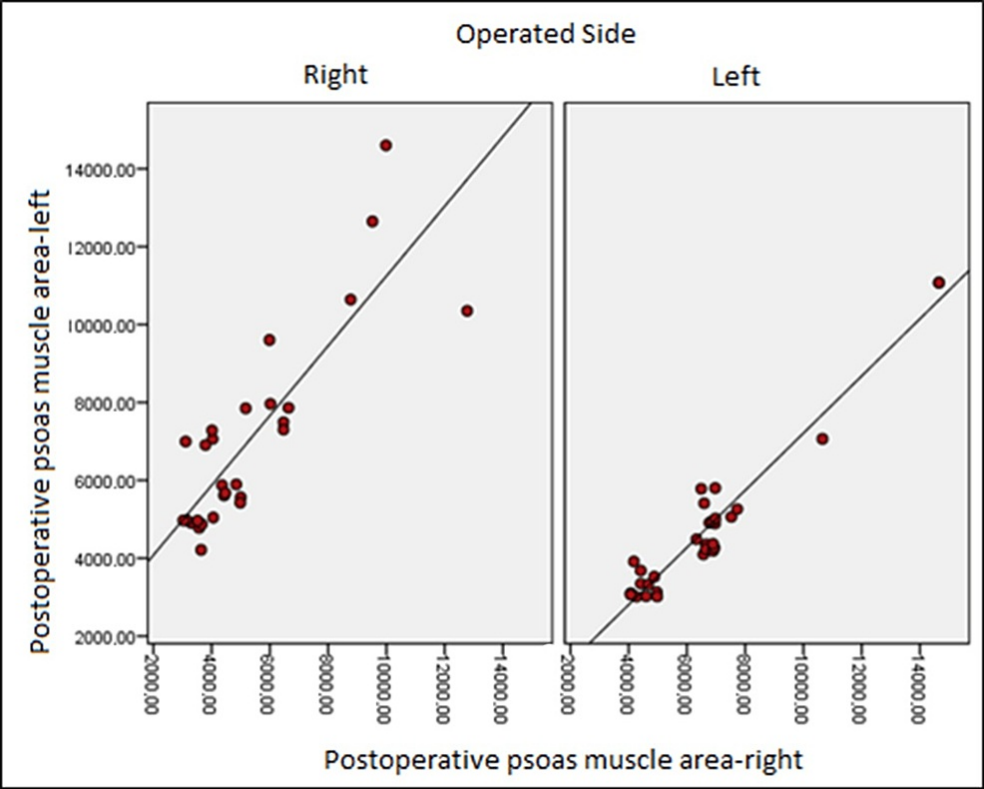
Distribution of postoperative psoas muscle area

Right preoperative psoas muscle area and left preoperative psoas muscle area values had a positive, 52.7%, and statistically significant connection in individuals who underwent right-side surgery (p:0.004; p0.05), respectively, and there was a statistically significant link (p: 0.000; p 0.05) between the right postoperative psoas muscle area and left postoperative psoas muscle area values at the level of 81.5% (Table 3) (Figures 2, 3).

**Table 3:** Analysis of correlation in the preoperative and postoperative psoas muscle areas *p<0.05; r results of Pearson correlations

Operated Side		r	p
Right	Preoperative psoas muscle area (Right-Left)	0.527	0.004*
	Postoperative psoas muscle area (Right-Left)	0.815	0.000*
Left	Preoperative psoas muscle area (Right-Left)	0.667	0.000*
	Postoperative psoas muscle area (Right-Left)	0.839	0.000*

In those who underwent left-side surgery, there was a positive relationship between right preoperative psoas muscle area and left preoperative psoas muscle area values at the level of 66.7%, and there was a statistically significant relationship (p:0.000; p 0.05), as well as a positive relationship between right postoperative psoas muscle area and left postoperative psoas muscle area values at the level of 83.9% (Table 3) (Figures 2, 3).

## Discussion

In this study, we observed that the operated and unoperated sides of the psoas muscle experienced considerable muscular atrophy after total hip arthroplasty at the L3-4 level. This result was consistent with earlier research [1,4,8] on gluteal muscles following unilateral total hip arthroplasty. There was a correlation between the atrophy of the psoas muscles on the operated and unoperated sides.

Few studies have examined muscle atrophy following unilateral hip implantation over time. A modest study of 22 patients concluded that the hip muscles permanently atrophy two years following a unilateral hip implant, as shown by cross-sectional area, a measurement of fat infiltration [4]. Mak et al. also found that atrophy in the psoas muscle was permanent in their study and stated that there was no significant difference between the operated and non-operated sides [10]. The current study found a significant decrease in psoas muscle area on both the operated and non-operated sides. We think that the reason for this is that although the patients have a pain-free life after the surgery, the atrophy in the muscles continues because they cannot fully return to high-level vital activities as in the early ages. However, a slightly larger study of 40 patients showed a significant increase in implant-side cross-sectional area in all gluteal muscles over two years (mean four years) after unilateral hip implant compared to initial postoperative CT. However, the gluteal cross-sectional area on the implant side remained considerably smaller than on the non-operative side [9]. More research involving larger patient populations is required to track changes in muscle cross-sectional area over time.

Colleagues have previously discussed leg dominance, which has been theorized to be the reason for the asymmetry in gluteal minimus muscle volume in people with degenerative hip joint disease [1]. Recent findings from a thorough review indicate that hip OA is not more common in the non-dominant leg than in the dominant leg [9]. Although the leg dominance of the patients in our study was unknown to us, our findings showed no significant correlation between the side of the implant and the cross-sectional area difference, indicating that leg dominance may not significantly contribute to the atrophy of the psoas muscle following hip replacement [11,12].

We demonstrated that when all other parameters are identical, the degree of hip OA on the non-operative side and the quantity of fat infiltration on the implant side greatly affect the amount of muscle atrophy. This highlights how crucial it is to consider all these aspects simultaneously when creating patient rehabilitation programs.

The healthy leg is typically weakened in patients with unilateral hip osteoarthritis as a result of their immobility, and after surgery, an equivalent recovery in both limbs should be anticipated [13,14]. Postoperatively, we observed a reduction in the psoas muscle area of the operated leg and radiological density in the unoperated limb. In the healthy limb, which served as the control group, we also found a radiological decrease in the psoas muscle area. We needed to research a set of external control people that were matched for age and sex to conduct an unbiased comparison. However, this experimental design would be severely constrained by the need for many healthy volunteers to acquire statistical power and the high cost of the necessary radiological measurements. The amount of atrophy in the operated limb and the amount of atrophy in the healthy limb were shown to be significantly correlated in the current study. An explanation is due to the healthy limb's lack of development. Even after a successful THR, patients could not move all that much. Alternately, it might be hypothesized that throughout the painful osteoarthritis-preoperative years, the muscles of the healthy limb were relatively stressed.

Injury to the THR during surgery may change how quickly muscles heal after surgery. After THR and conventional rehabilitation, Suetta et al. found a 13% and 9% decrease in quadriceps cross-sectional area at one and three months, respectively [15]. These early data points were absent from our current analysis. The surgical approach may impact both surgical trauma and rehabilitative issues. All of our patients had surgery using the posterior approach, during which the motor nerve (branch of the inferior gluteal nerve) and gluteus maximus were bluntly detached. This may imply that in patients with hip osteoarthritis, the location of the incision does not affect how well the atrophied muscles repair. Long-term research on various surgical methods, including less invasive ones, may provide insight into this problem.

The muscular changes we find could impact a person's quality of life, balance, and gait. The purpose of a total hip replacement is to relieve pain and restore patients' good functional motions to enhance their quality of life. Further evaluation of patients' functional scores (hip scores) before and following surgery, along with measurement of psoas muscle mass, may aid in identifying a patient population that may need specialized physical therapy regimens. To enhance post-operative function and mobility, early detection of psoas muscle mass decrease may enable a more proactive psoas strength development program.

There are some limitations to our retrospective study. As a retrospective study, MRI was not planned in the same way, and the timing of MRI may have varied. One of the most important limitations of the study is that the patients had lumbar MRIs before and after surgery. This shows that patients have both hip and lumbar problems. Therefore, this study shows that the decrease in psoas muscle volume is not due to osteoarthritis alone. The patients' neurological and cardiovascular problems were not taken into consideration. Patients were not examined for preoperative problems; pre- or postoperative therapy was not provided, and we were unsure how long arthritic patients had been experiencing symptoms. The influence on patients' hip movement, gait, and quality of life has to be evaluated clinically in relation to our findings.

## Conclusions

In the present study, we found that psoas muscle mass decreased significantly on both the operated and non-operated sides. The muscle changes we found can affect a person's quality of life, muscle strength, balance, and gait. Further evaluation of patients' functional scores (hip scores) before and after surgery and measurement of psoas muscle mass may help identify a group of patients who may need specific physical therapy regimens. Early detection of psoas muscle mass loss may provide a more proactive approach to increasing psoas strength to improve function and mobility after surgery.
